# The Bacteriology of Some Suppurations Complicating Pulmonary Disease
1Read before the Bristol Medico-Chirurgical Society, February 8th, 1899.


**Published:** 1899-03

**Authors:** J. O. Symes

**Affiliations:** Bacteriologist to the Bristol Royal Infirmary.


### THE BACTERIOLOGY OF SOME SUPPURATIONS COMPLICATING PULMONARY DISEASE.[1]

BY

J. O. Symes, M.D., D.P.H.,

*Bacteriologist to the Bristol Royal Infirmary.*

---

**Pneumonia.**—The first series of cases to which I wish to draw attention is that in which, together with either lobar or catarrhal pneumonia, there existed one or more foci of suppuration in other parts. I have examined seven such cases. In four of these the lung was apparently the starting point of the infection.

*Case* 1.—Aged 3 years. Was admitted suffering from lobar pneumonia, during which cerebral symptoms developed. At the necropsy there was well-marked croupous pneumonia and pleurisy, together with suppurative infiltration of the lateral ventricles of the brain, without general meningitis. Friedländer's pneumo-bacillus was isolated from the lung and from the pus on the ependyma.

*Case* 2.—Aged 21 years. Admitted with broncho-pneumonia, which terminated fatally. The necropsy showed suppurative peritonitis, and pus in the right middle ear, in addition to broncho-pneumonia. The suppurative lesions were not recognised during life. Pneumococci were present in the pus from all three situations.

*Case* 3.—Aged 6 years. Clinically there were signs of broncho-pneumonia and meningitis, and the *post-mortem* examination showed catarrhal patches in both lungs and pus in both tympanic cavities. There was no meningitis. Pneumococci were detected in the pus from the ears, in the consolidated areas of lung-tissue, and in the spleen.

*Case* 4.—Admitted with lobar pneumonia of left lung, and died soon after admission. In addition to pleuro-pneumonia, suppurative pericarditis was found at the *post-mortem* examination, and bacteriological examination showed the existence of pneumococci in lung, in pericardium, and in spleen.

These cases, especially those in which the organism was cultivated from the spleen, illustrate the fact that pneumonia is frequently a general infection. Whether it is always so one cannot say with certainty. It is interesting in this connection to note that in severe cases the pneumococcus can not infrequently be demonstrated during life in the blood. Thus Kohn isolated the pneumococcus from the blood in nine out of thirty-two

[1] Read before the Bristol Medico-Chirurgical Society, February 8th, 1899.

cases of pneumonia. Of these nine positive cases seven died, and the other two were complicated respectively by empyema and pneumococcus suppuration. Of the twenty-three negative cases only five died, so that one may conclude that the presence of the pneumococcus in the blood of a patient suffering from pneumonia is to be regarded as a bad prognostic indication.

There is evidently scope for *ante-mortem* as well as *post-mortem* blood examination in cases of pneumococcus infection.

A point of practical importance suggested by the cases just quoted is the necessity for examining the ears in all cases of pneumonia with cerebral symptoms, and if signs of pus be detected of evacuating it by incising the membrana tympani.

The following case might perhaps have been fairly classed with the four preceding :—

A child of 15 years was admitted in a typhoidal state, with delirium, and other well-marked cerebral symptoms. There was no optic neuritis, and Widal's test was negative. Later there were signs of broncho-pneumonia, and death ensued. The *post-mortem* examination showed a general pneumococcus infection, there being broncho-pneumonia, suppurative cerebral and spinal meningitis, and pus in tympanic cavities. The right membrana tympani was perforated. The history showed that when eight years old there had been suppuration of the right middle-ear and perforation of the membrane. The question then arises: Was the right tympanic cavity the starting point of the infection ?

In three out of the first four cases in which otitis media existed there seems little doubt but that this was secondary to the lung condition.

Personally, I do not know whether the pneumococcus is found in *chronic* middle-ear discharges; but as it is normally present in the mouth and pharynx, it is not unlikely that it should reach the ear and, exciting a suppuration, be the starting point of a general infection. On the other hand, it is equally possible that the old ear-mischief was again lit up by a pneumococcal infection from the lung. Again, the case may have been one of simple cerebro-spinal meningitis, a disease of which the pneumococcus is frequently the exciting cause.

The danger to life of **middle-ear suppuration**, although no symptoms of mastoid implication can be clinically detected, is well illustrated by the following cases :—

*Case* 1.—Admitted with membranous stomatitis and tonsillitis. Died of broncho-pneumonia. Bacteriological examination of the membrane before and after death showed staphylococcus albus and aureus only. Cultures taken at the *post-mortem* examination from consolidated areas of the lung and from pus in the right tympanic cavity showed streptococci. There had been discharge from the right ear for six months previously, and one may assume that this was the exciting cause of the streptococcic pneumonia.

*Case 2.*—Aged 5 years. Was admitted with discharge from right ear of many months' duration. No mastoid tenderness, but broncho-pneumonia and signs of meningitis. The necropsy showed pus in right tympanic cavity, extra-dural abscess, sinus thrombosis and multiple suppurative centres in the lung, all of which gave rise on culture to abundant growth of streptococcus longus. There was no meningitis.

In acute cases of middle-ear disease with **mastoid implication** calling for operative interference, I have on several occasions found the streptococcus to be either the predominant organism, or to be present in pure culture. It apparently is the most common causative agent, whilst second comes the pneumococcus, staphylococcus being a secondary infection. It would be of great interest to determine whether the streptococcus is constantly present in chronic ear discharges. In the throat the streptococcus appears to be normally present, at times attaining to a high state of virulence, and exciting tonsillitis and stomatitis. It may be that it undergoes similar changes of virulence in the ear, or possibly when introduced from without it finds in the chronically inflamed tympanic cavity a suitable nidus, and excites a suppuration of more than ordinary intensity.

The last case of pneumonia with suppuration which I purpose mentioning occurred in a case of enteric fever.

During life, in addition to the regular symptoms of the disease, there was marked deafness and retraction of the head, together with signs of pneumonic consolidation. At the necropsy the right tympanic cavity was full of pus, there was a septic thrombus in the kidney, and broncho-pneumonia. Bacillus coli was isolated from kidney, ear, and lung. As the necropsy was made thirty-six hours after death, it is doubtful whether this result has any true value. In abdominal diseases, and especially in typhoid fever, the bacillus coli invades the tissues very quickly after death, and so rapidly does it multiply that any other organisms present are apt to be crowded out and disappear. The case, however, serves to illustrate a point which I have verified at other *post-mortem* examinations, viz., that this organism is not infrequently the exciting cause of broncho-pneumonia.

**Pleural effusions** may be divided into three classes: (i.) Mechanical, such as occur in cardiac disease, in some forms of renal disease, and in malignant implication of the pleura. (ii.) Serous effusions of microbic origin. (iii.) Purulent effusions of microbic origin.

The only simple effusion I have examined was taken from a patient suffering from malignant disease of the lung. The fluid was sterile. At the *post-mortem* examination secondary implication of the pleura was discovered. The commonest exciting cause of non-purulent pleural effusion is undoubtedly the tubercle bacillus; fully 75 per cent. of so-called "idiopathic" pleurisies having this origin. Unfortunately it is seldom possible to demonstrate tubercle bacilli by centrifugalisation and film preparations, but if large quantities of the fluid (10 c.c.) be inoculated into guinea pigs positive results will be obtained in a large proportion of cases which would otherwise be styled "rheumatic" or "idiopathic."

Streptococci and pneumococci are also capable of exciting pleurisy, often as a primary disease, and further, if the conditions be such as to permit of the growth of these micro-organisms, the effusion will become purulent.

Tubercular effusions seem less liable to become purulent. Of 109 cases of empyema collected by Netter, twenty-nine were associated with pneumococcus, three with pneumococcus and streptococcus, forty-eight with streptococcus, fifteen fœtid cases with various saprophytic organisms, and only six with the tubercle bacillus.

I have had the opportunity of making a bacteriological examination in seven cases of empyema. In three the pneumococcus only was found, in one the pneumococcus with staphylococci and streptococci, in two staphylococcus (one aureus, one albus), and in one tubercle bacillus and staphylococcus albus.

The pneumococcal cases all followed attacks of pneumonia.

*Case* 1.—Aged 2 years and 5 months. Had broncho-pneumonia, and subsequent empyema, which was drained. The case terminated fatally, and at the *post-mortem* examination there was found an abscess at the base of the lung on the affected side. The pus from the empyema during life and from the abscess after death showed pneumococci.

*Case* 2.—Aged 4 years. Said to have had pneumonia one month previously. Pus from empyema showed pure culture of pneumococcus.

*Case* 3.—Aged 8 years. Lobar pneumonia four weeks previous to operation for empyema. The effusion showed presence of pneumococcus.

*Case* 4.—Empyema opened six months previously, but still discharging. The pus showed capsulated diplococci, probably pneumococci, also staphylococcus pyogenes aureus.

It is noteworthy that all these pneumococcal cases required operation and drainage. It has been held by some observers that a pneumococcal empyema may frequently be cured by simple aspiration, but this is a point on which there is likely to be much difference of opinion. There can, however, I think, be no doubt that the pneumococcus infection is less dangerous than that due to streptococcus or staphylococcus.

Both the staphylococcus suppurations included in the seven cases were fatal.

*Case* 1.—Had a history of long-standing pulmonary trouble, dating from an attack of bronchitis thirty years previously. The *post-mortem* examination showed that the left pleura was converted into thick calcified plates, and the contained pus showed staphylococcus pyogenes albus.

*Case* 2.—Aged 3 months. The empyema was preceded by a broncho-pneumonia. The pus drained off during life showed staphylococcus pyogenes aureus, and the same organism was present in the pus from an abscess cavity in the lung of the affected side, as discovered after death.

It is doubtful whether the following should be classified as a case of empyema :—

*Case* 5.—Clinically there were signs of fluid at one base, but the exploring needle only brought away thick caseous material, which when examined microscopically was found to contain cocci (which on culture proved to be staphylococcus pyogenes albus) and tubercle bacilli. Such a condition may be regarded as a tubercular pleuritis rather than empyema, but the case serves to raise the question as to whether it is advisable to operate when it is known that the empyema is of tubercular origin.

In conclusion, I may say that the manner in which organisms reach the pleuritic cavity is a matter for conjecture. It would not seem probable that either pleurisy or empyema can be simple primary affections. In health the air in the lungs must be as a rule free from microbic life, or if organisms be present we should not, judging from analogy, expect them to have the power of penetrating a healthy pleura. On the other hand, in diseased conditions of the lungs the neighbouring pleura will certainly suffer, and through a damaged membrane the organisms may pass out just as does the bacillus coli through an injured gut wall. Probably exposure to cold has this injurious effect upon the pleura and thus predisposes to pneumonia and pleurisy.

Our present knowledge of the lesions excited by the pneumococcus and its general infectivity, point to the unsatisfactory nature of our present methods of treatment and to the need for an efficient curative serum.

---

In the discussion which followed Dr. MICHELL CLARKE said that in cases of empyema information as to the micro-organism concerned might be got from the examination of coverslip preparations. He had not found in four cases of empyema due to the pneumococcus, that simple aspiration cured the patients. They all required incision later. He mentioned a case in which the diphtheria bacillus was abundantly present in the sputum, and in which there were symmetrically seated patches of consolidation in the apex of the lower lobe. There were no throat symptoms. He also alluded to the cases of disseminated broncho-pneumonia which followed influenza.—Dr. CAVE spoke of the value of the bacteriological examination of the blood and of the cerebro-spinal fluid in infection of the system and of the central nervous system in cases of pneumonia; also of the frequent biological association of staphylococci and tubercle bacilli in pleural effusion, so frequent that the demonstration of the former may justifiably excite suspicion of the essentially tubercular origin of the effusion. He also referred to Debove's demonstration of a substance analogous to tuberculin in tubercular effusions, and of his method of demonstrating its presence by injecting tuberculous guinea-pigs and producing a febrile reaction.—Dr. JAMES SWAIN said that suppurative processes produced by the pneumococcus somewhat resembled similar conditions caused by the tubercle bacillus. It was well known that tubercular abscesses rarely needed drainage, and there were also pneumococcal abscesses which had apparently been cured by evacuation without drainage.—Mr. ROGER WILLIAMS thought it remarkable that the pleural fluid was sterile in the only case associated with pulmonary malignant disease, as malignant tumours of any size nearly always teem with bacteria, etc., especially when in direct communication with the external atmosphere. Moreover secondary tumours also nearly always carry microbes with them, and as in this case there was secondary deposit in the pleura, it would seem that the pleural fluid was very likely to have been exposed to infection.

